# A Narrative Review of the State of the Art of CCR4-Based Therapies in Cutaneous T-Cell Lymphomas: Focus on Mogamulizumab and Future Treatments

**DOI:** 10.3390/antib13020032

**Published:** 2024-04-22

**Authors:** Corrado Zengarini, Alba Guglielmo, Martina Mussi, Giovanna Motta, Claudio Agostinelli, Elena Sabattini, Bianca Maria Piraccini, Alessandro Pileri

**Affiliations:** 1 Department of Medical and Surgical Sciences (DIMEC), University of Bologna, 40138 Bologna, Italy; corrado.zengarini@studiounibo.it (C.Z.);; 2 Dermatology Unit, IRCCS Azienda Ospedaliero-Universitaria di Bologna, 40138 Bologna, Italy; 3 Institute of Dermatology, Azienda Sanitaria Universitaria Friuli Centrale (ASUFC), 33100 Udine, Italy; 4 Division of Haematopathology, IRCCS Azienda Ospedaliero-Universitaria di Bologna, 40138 Bologna, Italy

**Keywords:** CCR4, CTCL, lymphoma, cutaneous, skin, oncology, chemokine, inflammatory, mediators, protein

## Abstract

The CCR4 receptor is a pivotal target in cutaneous T-cell lymphoma (CTCL) therapy due to its role in impairing immune responses against malignant T-cells and expression profiles. Monoclonal antibodies like mogamulizumab effectively bind to CCR4, reducing tumour burden and enhancing patient outcomes by inhibiting the receptor’s interaction with ligands, thereby hindering malignant T-cell migration and survival. Combining CCR4 antibodies with chemotherapy, radiation, and other drugs is being explored for synergistic effects. Additionally, small-molecular inhibitors, old pharmacological agents interacting with CCR4, and CAR-T therapies are under investigation. Challenges include drug resistance, off-target effects, and patient selection, addressed through ongoing trials refining protocols and identifying biomarkers. Despite advancements, real-life data for most of the emerging treatments are needed to temper expectations. In conclusion, CCR4-targeted therapies show promise for CTCL management, but challenges persist. Continued research aims to optimise treatments, enhance outcomes, and transform CTCL management. This review aims to elucidate the biological rationale and the several agents under various stages of development and clinical evaluation with the actual known data.

## 1. Introduction

Cutaneous T-cell lymphoma (CTCL) is a heterogeneous group of haematological cancers that primarily develops in the skin and is derived from T-cells. This definition accounts for several neoplasms with different prognoses and aetiologies, including primary cutaneous anaplastic large cell lymphoma, subcutaneous panniculitis-like T-cell lymphoma, adult T-cell leukaemia/lymphoma, primary cutaneous gamma-delta T-cell lymphoma, and also the extranodal NK/T-cell lymphoma, which represent approximately 75–80% of all primary cutaneous lymphomas [1]. However, mycosis fungoides (MF) and Sezàry syndrome (SS) are the most studied since their prevalence ranges from 60 to 80% in the population affected by CTCLs [2], usually present in males in their sixth decade [3,4].

MF is characterised by indolent behaviour in the early stages, while advanced phases are regarded as an aggressive disease like SS [5,6,7,8,9]. Based on such clinical behaviour, it is unsurprising that skin-directed therapies are recommended in early MF, while systemic drugs are warranted in SS and advanced MF [10]. However, despite scheduled therapies, the clinical outcome of advanced MF and SS has been invariably bad, with an estimated overall survival (OS) ranging from 4.7 to 1.4 years, according to the literature data [11,12,13,14].

A ‘revolution’ in CTCL treatment has started in the last few years. Indeed, the therapeutic armamentarium has been implemented, and an approach has been formed based on the destruction of highly proliferating cells (i.e., the action of chemotherapy). The goal of the newly available drugs is not merely based on the induction of the apoptosis of the neoplastic cells, but it is also found on the reawakening of the host immune system to contrast CTCL [5,6,7,9,15,16,17,18]. 

Indeed, besides the well-known chemotherapeutic agents (both single agents and polychemotherapy regimens) that present various limitations in clinical practice (patient immunodepression, cardiotoxicity, palmoplantar erythrodysesthesia, pancytopenia, and unfit patients due to co-morbidities) [13,14,19,20,21] and limited efficacy (time-to-next treatment range from 1.8 to 4 months [22,23,24]), new drugs have been developed; to date, more treatments are under investigation in clinical trials. 

Among the forerunners of the new generation of target therapies that exert their action by binding with molecules mainly or exclusively expressed by CTCL cells, mogamolizumab seems to be one of the most promising.

It exploits the chemokines’ different expression profiles possessed by cutaneous lymphomas compared to those in a non-tumoral environment.

Chemokines are a type of cytokines responsible for the chemotaxis of leukocytes. There are four different classes of chemokines, divided on the molecular basis of the first two cysteine groups in their structure: α-chemokines (CXC), β-chemokines (CC), γ-chemokines (C), and δ-chemokines (CX3C). 

Of these groups, CXC- and CC-related receptors (with the added suffix R to the terminology) are widely expressed in CTCLs [25,26,27].

More specifically, CCR3 has been found to be well expressed in CD30^+^ skin lymphoma [28], CCR7 seems to promote nodal infiltration and metastasis [29], CXCR4 has been found to be related in skin homing [30], and CCR10 appears to be involved in malignant T-cell trafficking [31], but among all of these, CCR4 is found to be widely expressed in tumoral CTCLs cells [32,33].

In concert, it is easy to think how such different expression levels can be exploited as a selective target and to block the mechanisms underlying this expressive model to treat the disease.

## 2. The CCR4 Receptor 

**Figure 1:**
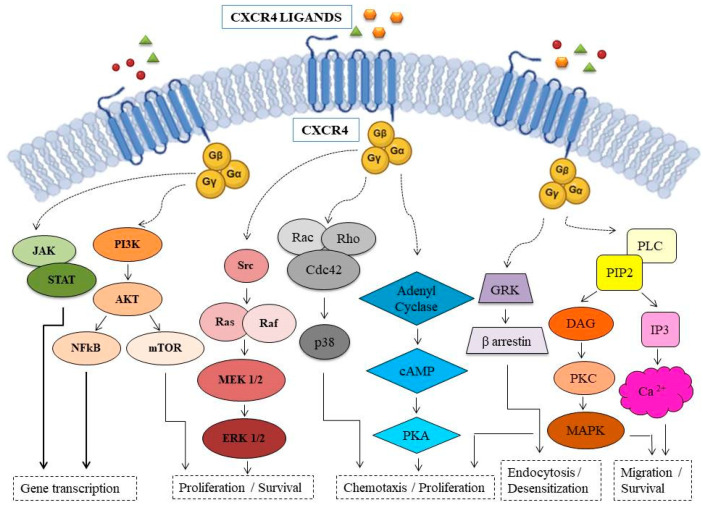
Schematic representation of the molecular pathways activated by CXCR4.

The CCR4 (β-chemokine/C-C chemokine receptor type 4) is a cell surface receptor encoded by its related gene and is also a cluster of differentiation (CD194) [34]. It is a G-protein-coupled receptor (GPCR) for signal transduction, causing cell activation and chemotaxis (Figure 1).

The following CC chemokines activate it: CCL2 (Monocyte Chemoattractant Protein-1—MCP-1), CCL4 (Macrophage Inflammatory Protein 1—MIP-1), CCL5 (Regulated upon Activation, Normal T-Cell Expressed and Presumably Secreted—RANTES), CCL17 (Thymus Activation-Regulated Chemokine—TARC), and CCL22 (Macrophage-Derived Chemokine) [35].

CCR4 is expressed mainly by lymphocytes [36], mainly on Th2 lymphocytes and is upregulated by T-cell receptor activation. Specifically, Th2-type CD4^+^ T-cells, which are involved in allergic inflammatory disorders such as asthma, atopic dermatitis, and allergic rhinitis, express CCR4. On the other hand, the expression of CCR4 on Th1 cells, which are usually involved in cell-mediated immunity against infection, is related to a different immune mechanism and is relatively depleted [18,37,38]. The effectiveness of anti-CCL17 and anti-CCL22 antibodies in murine asthma models supports and strengthens a prevalent function for CCR4 on Th2 cells; as Th1 and Th2 responses have opposing functions, CCR4 antagonists act as adjuvants to steer the immune response toward a Th1-type response [39,40,41].

Tregs and plasmacytoid dendritic cells (pDCs) also express CCR4. Some reports explored its importance in the trafficking of dendritic cells [42]. Specifically, Tregs keep DCs immature, making them ineffective at stimulating T-cell responses. Given that CCR4-mediated migration in response to chemokines produced by DCs is essential, CCR4 antagonism, implicated in DC-T cells, may improve immunological responses by disrupting Tregs’ inhibitory role. Moreover, CCR4 expression varies among different T-cell effector subsets. Therefore, CCR4 antagonists may produce various types of immunological responses. 

CCR4 is often expressed on leukemic cells in CTCL [43]. Notably, CCR4 expression on leukemic cells in CTCLs has been shown to play a pivotal role in the pathogenesis of these malignancies [33]. Among targeted therapies, mogamulizumab, a humanised monoclonal antibody directed against CCR4, demonstrated effectiveness in CTCLs [44]. 

CCR4 is a therapeutic target for different malignancies due to its role in immune cell recruitment in the tumour microenvironment. On the other hand, CCR4 underexpression is observed in autoimmune diseases, including multiple sclerosis [36,45]. 

This multifaceted involvement of CCR4 in various diseases highlights its significance in medical research and the potential for targeted therapies in disorders ranging from haematological malignancies to autoimmune and allergic conditions.

### CCR4 in Cutaneous Cell Lymphoma Development

Due to its role in malignant lymphocyte chemotaxis, CCR4 is expressed in MF (including CD8^+^ subtypes), SS, and other lymphomas [46], such as T-cell leukaemia/lymphoma (ATLL) [47]. 

CCR4 is expressed by both tumour cells and microenvironment cells. In a subset of individuals with CCR4^+^ ATLL, the tumour cells may act as regulatory T-regs cells, promoting tumour immune escape. Moreover, specific ligands for CCR4, produced by the tumour cells and the tumour microenvironment cells, attract CCR4^+^ Treg cells, leading to tumour immune escape in different types of cancers [48].

Hence, the potential of addressing CCR4 is to use it as a selective target and inhibit it to re-establish the immune response.

## 3. The Developed and Under-Investigation Treatments to Address CCR4

Several therapies interacting with CCR4 receptors have been studied or are currently under investigation Table 1.

We focused on mogamolizumab, a humanised monoclonal antibody directed against CCR4 with potential anti-inflammatory and antineoplastic activities.

Regarding the small molecules, many compounds have common characteristics and functions as GPCR ligands, including the biphenyl tetrazole moiety [36,49], a vaccine adjuvant. 

Researchers have studied several antagonists classified as small soluble molecules that can interact with CCR4. These antagonists have been classified into two main groups according to their binding with CCR4 sites [50]: Class I antagonists bind CCR4 extracellularly, while Class II antagonists bind CCR4 intracellularly.

They include drugs studied in vitro, in a murine model, and in vivo such as compound **22** (Bristol-Myers-Squibb), compound **8c** (Astellas, Tokyo, Japan) [51], RS-1748 (Daiichi Sankyo, Tokyo, Japan) [52], Zelnecirnon (Rapt Therapeutics, San Francisco, CA, USA) [53], GSK2239633 (GlaxoSmithKline, Brentford, UK) [54], AZD-1678 and AZD-2098 (AstraZeneca, Cambridge, UK) [55], SP50 (Matthew N. Davies et al.) [49,56], and related compounds, C021 (Lett et al.) [57], K777, CCR4 antagonist 18a, and CCR4-351. Most of these molecules have been investigated in the contexts of allergies, Th2-mediated infections, autoimmune diseases, and vaccinations.

Of the entire panorama of soluble molecules, we report below those that achieved some results in, at least, in vitro stage research and those with data reported on the Medline database. Finally, we discuss the reported data on CAR-T (Chimeric Antigen Receptor T-cell therapy) targeting CCR4 [58].

**Table 1:** Approved or under-investigation CCR4 receptor therapies.

Drug Name	Type of Drug	Approved Status	Indication	References
Mogamulizumab	Monoclonal antibody	Approved for CTCLs	Cutaneous T-cell lymphoma (CTCL)	[59,60,61,62]
Compound **22**	Small-molecular antagonist	Pre-clinical investigation	Investigated for allergies, Th2-mediated infections, autoimmune diseases, and vaccinations	[57,63]
Compound **8c**	Small-molecular antagonist	Pre-clinical investigation	Investigated for acute dermatitis treatment	[51]
RS-1748	Small-molecular antagonist	Pre-clinical investigation	Investigated for airway inflammation	[52]
Zelnecirnon	Small-molecular antagonist	Clinical trial	Investigated for atopic dermatitis	[53]
GSK2239633	Small-molecular antagonist	Clinical trial	Investigated for asthma indication	[64,65]
AZD-1678	Small-molecular antagonist	Pre-clinical investigation	Investigated as CCR4 receptor antagonist candidate drugs	[55]
AZD-2098	Small-molecular antagonist	Pre-clinical investigation	Investigated as CCR4 receptor antagonist candidate drugs	[55]
SP50	Small-molecular antagonist	Pre-clinical investigation	Investigated for vaccinations	[49,56]
C021	Small-molecular antagonist	Pre-clinical investigation		[57]
K777	Small-molecular antagonist	Pre-clinical investigation	Investigated as antiviral agent	[66,67]
CCR4-351	Small-molecular antagonist	Pre-clinical investigation	Investigated for lymphoblastic and epithelial neoplasms with positivity for Epstein–Barr Virus	[68]
CCR4 antagonist 18a	Small-molecular antagonist	Pre-clinical investigation	Investigated as antiviral agent	[67]
C01 dihydrochloride	Small molecule	Pre-clinical investigation	Cutaneous T-cell lymphoma (CTCL)	[57]
CAR-T	Chimeric Antigen Receptor T-cells	Clinical trials for CTCLs	T-cell malignancies	[58,69]
Chloroquine/Hydroxychloroquine	Anti-malarial drug	Approved for autoimmune diseases, and investigational drug for CTCLs	Systemic lupus erythematosus, rheumatoid arthritis, porphyria cutanea tarda, q-fever, malaria, and inflammation	[70,71,72,73]

### 3.1. Mogamolizumab

Mogamulizumab (Codename KW-0761) is a defucosylated humanised monoclonal antibody against CCR4, and it exerts its antitumour action by binding CCR4 and consequently inducing the apoptosis of neoplastic T-cells, and by depleting immunosuppressive cells recruited by malignant counterpart, leading to an increase in host immune response against the disease [59,74,75]. The efficacy of mogamulizumab has been evidenced in the MAVORIC trial [60,61], an open-label, multinational, phase 3 randomised controlled trial that compared mogamulizumab with vorinostat, an oral histone deacetylase inhibitor, in relapsed or refractory CTCL patients. The trial showed that mogamulizumab presented a significant increase in progression-free survival (PFS) compared to vorinostat (hazard ratio: 0.53, 95% CI [0.41–0.69], *p* < 0.0001). Post hoc analysis and real-life clinical practice corroborated the MAVORIC trial data [44,59]. The drug was administered at 1 mg/kg intravenously weekly in the first 28-day cycle and then on days 1 and 15 of the subsequent cycles until disease progression was observed. 

#### 3.1.1. Real-Life Experience and Post Hoc Analysis

Beylot-Barry et al. [62] performed a post hoc analysis of the MAVORIC trial data, revealing a median time-to-global response (TTR) of 3.3 months. The times-to-compartmental response was 1.1 months in blood, 3.0 months in skin, and 3.3 months in lymph nodes. The parameters mentioned above were not estimable for visceral involvement. Beylot-Barry et al. [62] aimed to analyse overall response rate (ORR), progression-free survival (PFS), time-to-next-treatment (TTNT), and TTR, narrowing the cohort to MF patients treated with mogamulizumab and showing that, in MF patients, ORR, PFS, and TTNT were directly related to the blood involvement. The improvement estimated with ORR, PFS, and TTNT was higher in patients with a higher blood tumour burden. 

In the French group analysis, half of the patients exhibited a global response later than the three months reported in the MAVORIC trial (up to 8.7 months). Skin response occurred with a median time of 3.9 months, but half of the patients responded later (up to 13.2 months). The take-home message of the paper is that clinicians should wait at least five months before switching an MF patient from mogamulizumab to another drug, and patients should be informed of the possibility of a delayed therapeutic response. MF patients with a high blood tumour burden (i.e., B1 and B2 based on EORCT/CLTF staging) should be considered the best candidate for the treatment. A possible explanation of the better efficacy in those patients may be related to mogamulizumab’s better access to neoplastic cells in peripheral blood rather than in the skin, leading to a higher reduction in the global tumour burden as pointed out by the first post hoc analysis by Cowan R.A. et al. [44]. 

Furthermore, as observed by Horwitz S. et al. [60], mogamulizumab efficacy was not influenced by the number of prior-administered therapies, revealing it to also be an efficient treatment in heavily pre-treated patients. Patients with a higher risk of becoming non-responders were those who showed a loss of CCR4 antigen expression, as observed in 17 patients by Beygi S. et al. [76]. Moreover, the loss of CCR4 expression should discourage clinicians from treating such patients with CCR4 targeted therapies. Concerning mogamulizumab efficacy, Ohuchi et al. [77] hypothesized that serum CCL-22 levels may correlate with mogamulizumab response. Indeed, in the described cases, patients who responded to mogamulizumab presented a decrease in serum levels of CCL22, while no differences were identified in the serum levels of CCL17, CCL19, CXCL10, or CXCL13. Most CCL22-producing cells were CD-163+ tumour-associated macrophages surrounded by CCR4^+^ CTCL cells at immunofluorescence.

#### 3.1.2. Report on Combination Treatment

Real-world evidence on mogamulizumab revealed treatment efficacy in CTLC patients and highlighted the possibility of combination treatment with mogamulizumab and other drugs. 

Hisamoto T. et al. [78] proposed the combination treatment with mogamulizumab and bexarotene by describing a patient who achieved complete remission (CR) after the combination scheme. Also, Teoli et al. [79], despite the different mechanisms of action of these two drugs, reported promising results in patients treated with mogamulizumab and bexarotene. The authors specified that mogamulizumab was added after bexarotene began achieving a reduction in mSWAT score during follow-up. Teoli et al. [79]. enthusiastically highlighted that the response was maintained longer than expected. 

Only one paper has provided evidence on the efficacy of the combination of mogamulizumab with total skin electron beam treatment (TSEBT) [80]. In our opinion, such a combination approach may be promising and should be encouraged owing to the different actions of TESBT and mogamulizumab. Indeed, TSEBT may induce an increase in neo-antigens from the skin to blood. At the same time, mogamulizumab may provide a response in the blood compartment, leading to an immune response that empowers and reduces immunosuppressive cells. Fong et al. [80] observed a complete response in two SS patients after TSEBT + mogamulizumab. Interestingly, low-dose total skin electron beam therapy (LD-TSEBT) was given before mogamulizumab therapy. 

Other encouraging data have been recently provided by Rubio-Muniz C.A. et al. [81], who described seven SS patients treated with a combination of extracorporeal photopheresis (ECP) and mogamulizumab. In six skin-level instances, a partial response (PR) was observed, while in peripheral blood, five CRs and one PR were detected. 

Although the cohort of patients is limited in all the cases described in the literature, such preliminary data pave the way for combination treatment with mogamulizumab and other treatments, especially those known to exert their action by directly acting on neoplastic cells (like TSEBT) and/or also acting on the microenvironment or host immune system (like ECP or bexarotene). More cases are required to corroborate preliminary data, possibly with prospective multicentric studies. 

#### 3.1.3. Mogamulizumab-Associated Rash

Since the MAVORIC trial, mogamulizumab-associated rash (MAR) has been reported as the second most common adverse event [60,61]. Numerous reports in the literature have contributed to depicting such types of adverse events/effects [82,83,84,85,86,87]. Our group was the first to describe an MAR in 2018. The described patient presented with a skin eruption characterised by erythematous plaques on the face and the neck, which was regarded as a sort of a treatment-related pseudo lymphomatous reaction. Indeed, after the suspension of mogamulizumab, the lesions regressed.

Interestingly, the plaques simulated a relapse in SS, the disease that affected the patients. Clinical practice has improved our knowledge on MAR, and to date, four clinical patterns (folliculotropic MF-like scalp with alopecia, papules and/or plaques, photo-accentuated dermatitis, and morbilliform or erythrodermic dermatitis) have been described along with peculiar histological presentation (psoriasiform/spongiotic pattern, lichenoid/CD8^+^ interface presentation and granulomatous, with mixed pattern). In most of the paper, it is stressed that MAR is difficult to distinguish from a CTCL relapse at a skin examination. At the same time, immunohistology provides clues for MAR diagnosis (presence of peculiar infiltrate as mentioned above and the expression of pan-T-cell markers such as CD7 and polyclonal TCR) [82,83,84,85,86,87].

Apart from the most common MAR presentation, sporadic cases of MAR presenting as lupus miliaris facei [84], alopecic lesions [82,86], and sebaceous hyperplasia eruption [88] have been reported in the literature. All the reported experiences strongly agree that MAR development is more common in SS and related to a better clinical outcome [4,82,83,84,85,86,87,89,90,91]. Such a finding is not trivial and should be associated with a direct action on neoplastic cells (i.e., apoptosis of tumour cells) and the decrease in immune suppressive cells (Treg cells). Hence, MAR should not be considered a mere adverse event but an excessive activation of the immune system.

#### 3.1.4. Recent and Ongoing Clinical Trials

Since 2018, the year of publication of the MAVORIC study, further studies in addition to those already cited, both under controlled and real-world settings, have taken place to investigate the efficacy and safety of mogamolizumab in CTCLs and other solid tumours. The table shows the currently published results of the most recent and ongoing trials listed in the Medline database (Table 2).

### 3.2. Zelnecirnon (RPT193)

Zelnecirnon (code name: RPT193) is a molecule under investigation by RAPT Therapeutics, Inc. [53], for atopic dermatitis. In in vitro studies, it inhibited Th2 cell infiltration and chemotaxis [103] and reduced Th2-derived AD lesions in a murine model [104].

It is administered orally, and in the phase 1 study, the gene expressions of known biomarkers such as IL-19, CCL20, and CXCL1 were significantly downregulated in the RPT193-treated arm, along with IL-8, CCL19, CCR7, CCL2, IL-9, and the regulatory CTLA-4, also known to be related to CTCLs’ pathogenesis. The phase 2 study is currently ongoing [53].

There are no known in vivo or in vitro CTCL-related studies regarding this compound.

### 3.3. K777

K777 is a selective CCR4 antagonist featuring potent chemotaxis inhibition, is also an orally active and irreversible cysteine protease [105], is a CYP3A4 inhibitor, and is a broad-spectrum antiviral targeting cathepsin-mediated cell entry (showing efficacy against SARS-CoV and Filovirus [63]).

In the study of a compound that could inhibit Th2 lymphocyte chemotaxis for addressing allergic diseases, Sato et al. [64] have discovered in a binding assay using CCL17, a CCR4 ligand, the low effectiveness of the molecule [64].

In in vitro studies based on a chemotaxis assay of Hut78 cells, K777 showed a potent inhibitory activity for chemotaxis. The activity was higher than expected and more powerful than the classical competitive antagonist of a GPCR. It also effectively induced CCR4 internalisation without affecting CCR8 or CXCR4 chemotaxis [64,65].

Unfortunately, studies on this compound have remained at the pre-clinical stage and are very limited [106]. This is unfortunate given the high selectivity for CCR4 and the ability to induce internalisation, a factor that could easily be exploited in treating CTCLs.

### 3.4. CCR4 Antagonist 18a

Compound [8,9,10,11,12,13,14,15,16,17,18,19,20,21,22,23,24,25,26,27,28,29,30,31,32,33,34,35,36,37,38,39,40,41,42,43,44,45,46,47,48,49,50,51,52,53,54,55,56,57,58,59,60,61,62,63,64,65,66,67,68,74,75,76,77,78,79,80,81,82,83,84,85,86,87,88,89,90,91,92,93,94,95,96,97,98,99,100,101,102,103,104,105,106,107] (CCR4 antagonist 18a), not to be confused with another molecule also named 18a (in the literature also known as HIV-1 inhibitor 18A, studied for its capacity to inhibit HIV-1 entry in cells expressing CCR5 [70]), was discovered by Lena Shukla et al. [107]. It was found to have a high affinity for CCR4 and to induce the internalisation of about 60% of the CCR4 cell surface receptors, which is generally unusual for small molecules.

Recently, another study showed that it binds and inhibits CCR4 signal [106] better than HCQ without QT prolongation effects, along with K777 and mogamulizumab.

As for K777, the capacity of receptor internalisation could be exploited to target CTCLs, but further studies are still lacking.

### 3.5. CCR4-351

CCR4-351 is an orally active, potent, and selective CCR4 antagonist.

In both in vitro and murine models, Marshall et al. [71] have assessed how CCR4-351, combined with an anti-CPI drug, inhibits Treg migration and increases CPI antitumour effects compared to high and low CCR4 ligand expression samples, respectively. They found that T-regs migrations into the tumour environment led to a more rapid tumour expansion and that CCR4 blockage by CCR4-351 reduced T-regs migration and increased antitumour activity, supporting the clinical development of CCR4 inhibitors in combination with CPI for the treatment of cancer [71].

Another study assessed the capacity of CCR4-351 to exert antitumoral effects on lymphoblastic (Hodgkin lymphoma) and epithelial (nasopharyngeal carcinoma) neoplasms with positivity for Epstein–Barr Virus, showing how antagonism of the CCR4 receptor with this molecule may effectively activate the immune response against EBV+ tumours [72].

Studies on CCR4-351 remain limited and at the pre-clinical stage, with no data regarding CTCLs.

### 3.6. AZD-1678 and AZD-2098

AZD-1678 and AZD-2098 are two compounds that Nicholas Kindon et al. group (AstraZeneca) developed. Nicholas Kindon et al. [55] revealed that these compounds exhibit increased activity and reduced lipophilicity, achieving plasma protein binding of less than 99% of CCR4 receptors and showing good selectivity for CCR4 receptors without any agonist activity at concentrations up to 10 μM.

Biological assays confirmed the antagonist potency of these compounds in various cell systems, demonstrating a lack of agonist activity. In vivo studies showed notable anti-inflammatory effects in sensitised rats, presenting a dose-dependent reduction in histological correlates.

Future studies will provide further insights into these compounds, particularly their potential for CTCLs.

### 3.7. SP50 and Related Molecules

SP50 is a molecule developed by Matthew N. Davies et al. [49] from the initial work of Allen et al. [73]. Allen et al. initially found how substituted thiazolidinones could bind CCR4 with inhibitory capacity (compounds **90** and **91**). These compounds showed a strong chemotaxis inhibition capacity of Th2 cells. Subsequent studies from Matthew N. Davies et al. [49] led to the identification of optimised and more potent antagonists [56]. Among these compounds, SP50 was initially studied for its theoretical ability to enhance the immune system by downregulating T-regs in vaccination contexts.

In these in vitro studies, SP50 is dissolved in DMSO and mixed with various vaccines, including pFLAG CMV4 vectors expressing *M. tuberculosis* proteins (CMV1818c and CMV3812) and adenovirus type 5 expressing the 42 kDa region of merozoite surface protein-1 (Ad-MSP-1 42) from *P. yoelii* [49].

The experiments involve immunising mice with these vaccine/SP50 mixtures and assessing the immune responses. The study indicates that SP50, among other CCR4 antagonists, can enhance the immune response when combined with specific antigens by modulating the migration of regulatory T-cells and Th2 cells, thus influencing the overall immune response [49].

Bozza et al. [56] showed that SP50 can reduce tissue damage, eosinophil recruitment, and serum IgE levels and modulate mucine expression. In a pulmonary fungal infection experiment, improved lung histopathology was observed when it was administered, and fibrotic change was reduced during the infection. The compound’s influence on Th2 and Th17 cytokines suggests its potential in suppressing these immune responses. An increase in IL-10 and FoxP3+ cells indicates a shift toward a more tolerogenic state.

In conclusion, SP50 in Th2-driven infections seems to induce tolerance, evidenced by increased IL-10 levels and decreased Th2/Th17-associated cytokines. Elevated IFN-γ levels indicate a broader effect on the immune response. Additionally, there is a potential for enhancing CD8^+^ cytotoxic T-cell responses, implying applications in vaccination [56].

The results of these studies are limited to in vitro studies and murine models, but their premises are attractive for future applications for CTCLs.

### 3.8. Compound ***22***

Compound **22** is one of the most studied small soluble molecules [32,41,56,69,108,109,110] and described by Purandare et al. [111].

In one experiment involving CCR4-deficient mice, compound **22** impacted Treg cell recruitment, DC activation, and overall immune response.

Notably, compound **22** enhances antigen-specific humoral and cellular immune responses by inhibiting Treg cell recruitment to the muscle tissue. The findings suggest that the intramuscular administration of CCR4 antagonists may serve as a practical approach to enhance vaccine efficacy. The document also discusses potential discrepancies with another CCR4 antagonist and concludes with the implications for future vaccine development [112].

There is also a study regarding its ability to reduce immune activity against melanoma cancer. Contrary to what was seen for CTCLs [57], Kazuhiko Matsuo et al. [112] discovered that targeting CCR4 in mice affected by melanoma enhanced tumour growth and decreased Th17 cells in regional lymph nodes in tumour-bearing mice treated with Dacarbazine. Their findings indicate that CCR4 is critically involved in regional lymph node DC-Th17 cell interactions that are necessary for Th17 cell-mediated induction of antitumour CD8^+^ effector T-cells in mice bearing B16 melanoma. Although more research on the function of CCR4 in other types of tumours is necessary, it is crucial in determining the efficacy and safety of cancer immunotherapies that CCR4 activates antitumour immunity through Th17 in a mouse model of B16 melanoma [112].

Finally, even with this molecule, some results demonstrate how inhibiting CCR4 increases the immunological potential of vaccines. In a study by Shinya Yamamoto et al. [112], compound **22** increased the production of IL-4 and IFN-γ in CD4^+^ T-cells and the levels of IFN-γ in CD8^+^ T-cells and OVA-specific IgG responses. Lastly, OVA and compound **22**, administered intramuscularly, dramatically slowed the development of tumours expressing OVA. CCR4 is essential for recruiting Treg cells to the muscle tissue, and injecting CCR4 antagonists intramuscularly might be helpful to boost vaccination effectiveness [112].

However, these studies create some doubts on whether inhibiting CCR4 may not be a win–win strategy for tumours, given that the study by Kazuhiko Matsuo et al. [112] showed melanoma progression in treated mice. Studies on CTCLs are necessary to delve deeper into the pathophysiology of the case.

### 3.9. Compound ***8c***

Kazuhiro Yokoyama et al. [51] studied compound **8c** for its characteristics in a CCR4 antagonist series, based on a solid 3D affinity between the docking site of the CCR4 receptors of humans and a murine model and evaluated it as a treatment for acute dermatitis. The downregulation of Th2 inflammatory cells’ activity seemed to be responsible for the antioxidant and anti-inflammatory effect of compound **8c**, which was demonstrated by the oxazolone-induced contact hypersensitivity test.

The studies remained at the pre-clinical stages, and there are no data regarding its effects on CTCLs.

### 3.10. RS-1748 and Related Compounds

CCR4 antagonists RS-1748, RS-1154, and RS-1269 demonstrated the ability to effectively block the binding of CCL17 and GTPgammaS ligands to Chinese hamster ovary (CHO) cells that express CCR4 [52]. Additionally, at a 10 mg/kg dosage, RS-1748 prevented ovalbumin-induced airway inflammation in guinea pigs. These findings suggest that these compounds, and among them, RS-1748, might be a good starting point for creating an asthma treatment [52].

Further data are needed on their effects on cancers and lymphomas.

### 3.11. GSK2239633

GSK2239633, also known as compound **7r**, in a study by Procopiou et al. [54], and in an in vitro essay, inhibited TARC binding to CCR4 and also inhibited TARC-induced F-actin in isolated human CD4^+^ CCR4^+^ T-cells.

In a phase 1 clinical study, GSK2239633 was well tolerated and capable of inhibiting TARC by activating the CCR4 receptor [113].

At the maximum dosage level of 1500 mg, the peak inhibition of CCR4 by GSK2239633 in the blood (at 1 h) was less than 80% and less than 50% at 4 h post-dose. In any case, this molecule showed modest and saturable systemic capacity. Due to its poor exposure, this chemical was deemed unsuitable for future research as an anti-asthma agent and maybe also for other diseases due to its low target engagement in blood and short half-life [113,114].

This trial, one of the few to have reached human phase 1 in clinical research, poses some pharmacokinetic problems regarding the development of small, soluble molecular antagonists of CCR4, apparently very effective on a molecular level but may not be so in vivo [113,114].

### 3.12. C01 Dihydrochloride

C01 is a small dihydrochloride molecule developed by Cheryl Lee et al.’s group and has been developed to decrease tumour proliferation and volume in CTCLs [57].

In their pre-clinical studies, cancer weight was lower in treated groups than in the control group, among those with high Ki67 levels. CCR4^+^ cells were not drastically decreased, but large necrotic areas in treated tissue have been observed. Their results showed how C01 had inhibitory effects on CTCL cell proliferation, which may contribute to decreased tumour volume in xenograft CTCL mice [57].

More data on bigger groups are needed to confirm these results and to devise further developmental steps.

### 3.13. Hydroxychloroquine

Hydroxychloroquine (HCQ) is a long-standing drug with many in-depth and varied studies than those reported above for other molecules, primarily due to the speculation of its effectiveness in containing the SARS-CoV-2 infection. It is a racemic mixture consisting of R and S enantiomers with an aminoquinoline-like structure (chloroquine—CQ), both able to downregulate several pro-inflammatory cytokines and T-cell subsets. It acts through different mechanisms and is helpful for various pathologies.

Again, thanks to the study by Beck et al. [106] and the study resumed by Jin et al. [115], Hydroxychloroquine would appear to have some effect on the CCR4 receptor, probably due to its quinoline ring nitrogen, which may be able to interact with the receptor pocket [116].

However, the available data on HCQ related to cutaneous lymphomas utilized other approaches. Fauzi et al. [117] showed how HCQ and CQ induced apoptosis in ATLL [117]: both molecules restored the expression of p47, blocking autophagy inside affected cells and sequentially inhibiting the activation of the NF-κB pathway, leading to apoptosis in ATLL cell lines.

There has also been an isolated clinical case of treatment of CTCLs with HCQ [118], while it is also known that several case reports use the same substance to treat diverse forms of follicular mucinosiss [119,120].

In any case, these results regarding HCQ and CCR4 interactions were obtained mainly from in vitro studies that need to be confirmed in vivo along with the unknown implications regarding their potential impact on the pathophysiology of CTCLs.

### 3.14. CAR-T

Some authors have explored the possibilities of CAR-T [121] (Chimeric Antigen Receptor T-cell therapy) that targets the CCR4 receptor [58,122].

Perera et al. [122] showed that it is possible to create functional CCR4-redirected CAR-T (CCR4-CAR-T) cells; nevertheless, the impact of the fratricide effect and its connection to the function, phenotype, target sensing, transduction, expansion, and antitumour activity of CAR-T against CTCL cells remained unclear.

Keisuke Watanabe et al. [58] further explored this possibility, developing another CCR4-CAR-T against CTCLs. This last experiment revealed promising outcomes, demonstrating that CCR4-CAR-T cells exhibited notable lytic activity against primary CTCL cells. The study scrutinised CAR-T cells’ cytokine production upon stimulation with primary CTCL cells, employing intracellular staining and flow cytometry analysis.

Comprehensively, they found that anti-CCR4 CAR-T cells specifically suppressed Th2 and Tregs while sparing CD8^+^ and Th1 T-cells, despite the widespread belief that fratricide in CAR-T cells is harmful to anticancer capabilities. This experiment’s characteristics included high transduction efficiency, robust T-cell proliferation, and fast fratricidal elimination of CCR4-positive T-cells during CAR transduction and expansion. Additionally, mogamulizumab-based CCR4-CAR-T cells produced higher antitumour effectiveness and long-term remission in mice engrafted with human T-cell lymphoma cells.

To summarise, CCR4-depleted anti-CCR4 CAR-T cells have robust antitumour effectiveness, and the group emphasises their potential as a therapeutic intervention for T-cell malignancies.

Both studies were conducted in vitro [58,122], but the results were favourable and specific towards CTCLs that express this receptor, opening an exciting avenue for further potential therapy

## 4. Discussion

The comprehensive exploration of CCR4 in the context of CTCLs presented in this paper sheds light on the intricate landscape of therapeutic avenues and their underlying mechanisms. The investigation into the only registered drug in this category, mogamulizumab, has revealed promising efficacy and potential hurdles, particularly in terms of MAR, which has emerged as a significant concern in clinical trials.

Notably, the document highlights the significance of CCR4 antigen expression as a critical factor in predicting patient response to treatments. The loss of CCR4 expression has been identified as a potential indicator of non-responsiveness, emphasising the importance of patient stratification based on molecular characteristics. Furthermore, exploring combination treatments, such as mogamulizumab with bexarotene or TSEBT, presents intriguing possibilities for enhanced therapeutic outcomes, as seen in documented cases that achieved complete remission.

The emergence of novel compounds as soluble CCR4 antagonists such as 18a, K777, CCR4-351, and AZD-1678/AZD-2098 and their exploration for other diseases has introduced a myriad of possibilities for future therapeutic interventions. Each compound exhibits unique characteristics, such as high affinity for CCR4, receptor internalisation capabilities, and potential synergies with anti-CPI drugs, laying the foundation for a more nuanced approach to CTCL treatment. However, it is crucial to acknowledge the limited nature of pre-clinical studies conducted for most of these compounds, emphasising the need for further research and clinical validation.

Once the potential of targeting the CCR4 receptor for these pathologies has been explored, already known drugs, notably, HCQ, would appear to be able to modulate it. Its interaction with the CCR4 receptor, though primarily gleaned from in vitro studies, adds a layer of complexity in understanding its potential impact on the pathophysiology of CTCLs.

Moreover, CAR-T therapy emerges as a highly specific and selective armamentarium against T lymphomas expressing CCR4, with promising results that need to be confirmed using in vivo studies.

In conclusion, the current research has added numerous new molecules selective for CCR4 in the pipeline after mogamulizumab paved the way for an entirely new therapeutic class, and mogamulizumab is still, at the moment, the only drug with solid data available on its efficacy. As the field continues to evolve, researchers encourage a multidimensional approach, incorporating molecular markers, combination therapies, and innovative compounds to enhance the precision and efficacy of CTCL treatments. It calls for further collaborative efforts, prospective multicentric studies, and a deeper understanding of the underlying mechanisms to propel the field forward and ultimately improve outcomes for individuals affected by CTCLs.

## Figures and Tables

**Table 2:** Research string in Medline database ‘mogamulizumab’ for ‘Clinical trials’ article type from 2020 to 2024. ORR: overall response rate; OS: overall survival; DOR: duration of response; and TSEBT: total skin electron beam therapy.

Title	Author	Date	Research Field	Results	References
Real-World Treatment Patterns and Clinical Outcomes with Brentuximab Vedotin or Other Standard Therapies in Patients with Previously Treated Cutaneous T-Cell Lymphoma in the United States	Stefan K Barta et al.	2023	Real-world data; brentuximab–vedotin vs. other treatments (including mogamolizumab) in CTCLs	Favourable outcomes with BV vs. OST in patients with CTCL previously treated with ≥1 systemic therapy	[92]
Integrated analysis of phase 1a and 1b randomised controlled trials; Treg-targeted cancer immunotherapy with the humanised anti-CCR4 antibody, KW-0761, for advanced solid tumours	Kaoru Fujikawa et al.	2023	Phase 1a and 1b trials to examine the safety and efficacy of mogamolizumab in augmenting immune therapy response in solid cancers	Durable clinical response in some patients	[93]
An open-label, single-arm phase 2 trial of valemetostat for relapsed or refractory adult T-cell leukaemia/lymphoma	Koji Izutsu et al.	2023	Phase 2 trial enrolled patients with R/R aggressive ATL treated with valemetostat, with some of them pretreated with mogamolizumab	Patients pretreated with mogamulizumab had an ORR of 45.8% (four complete and seven partial remissions)	[94]
Impact of blood involvement on efficacy and time to response with mogamulizumab in mycosis fungoides and Sézary syndrome	Marie Beylot-Barry et al.	2023	Post hoc analyses were carried out using data from MAVORIC	Compared with vorinostat, superior results were seen for ORR, PFS, and TTNT in mogamulizumab-treated patients with MF	[62]
Adjusting for treatment crossover in the MAVORIC trial: survival in advanced mycosis fungoides and Sézary syndrome	Neil Hawkins et al.	2022	Post hoc analyses were carried out using data from MAVORIC	OS of mogamulizumab relative to vorinostat may be underestimated in MAVORIC due to the presence of crossover	[66]
Phase Ib study on the humanised anti-CCR4 antibody, KW-0761, in advanced solid tumours	Takuro Saito et al.	2021	Phase 1b trial to assess the efficacy of mogamulizumab as an immunotherapeutic drug in solid tumours	Mogamulizumab resulted in the depletion of Tregs in peripheral blood and potential immune responses	[95]
Depletion of central memory CD8^+^ T-cells might impede the antitumor therapeutic effect of Mogamulizumab	Yuka Maeda et al.	2021	Phase 1b study, the cohort analysis of patients with advanced CCR4-negative solid cancer	Mogamulizumab’s current doses may deplete effector components in immune therapy	[96]
Mogamulizumab in Combination with Nivolumab in Phase I/II Study of Patients with Locally Advanced or Metastatic Solid Tumors	David S Hong et al.	2022	Phase I/II study, multicentric, to assess mogamulizumab with nivolumab in solid tumours	Combination therapy did not result in enhanced efficacy	[67]
Lack of impact of type and extent of prior therapy on outcomes of mogamulizumab treatment in patients with cutaneous T-cell lymphoma in the MAVORIC trial	Steven Horwitz et al.	2021	Post hoc analyses were carried out using data from MAVORIC	ORR and DOR remained consistent regardless of the type of immediately prior therapy. Additionally, the immunomodulatory activity of the last prior therapy and time from prior treatment generally did not affect the ORR or PFS	[97]
Short-course IL-15 given as a continuous infusion led to a massive expansion of effective NK cells: implications for combination therapy with antitumor antibodies	Sigrid P Dubois et al.	2021	Phase 1 study analysis	Mogamulizumab could benefit from NK expansion induced by IL-15 administration	[98]
Acute and sub-acute toxicity profile of ultra-hypofractionated low-dose total skin electron beam with two 4 Gy fractions for cutaneous T-cell lymphoma.	Daniel Rolf et al.	2021	Prospective study	Ultra-hypofractionated low-dose TSEBT followed by systemic therapy, including mogamulizumab, seems to be a feasible alternative to the conventional fractionated TSEBT	[99]
Quality of Life Effect of the Anti-CCR4 Monoclonal Antibody Mogamulizumab Versus Vorinostat in Patients with Cutaneous T-cell Lymphoma.	Pierluigi Porcu et al.	2020	Multicenter phase 3 trial	The symptoms, functions, and overall QoLs of patients with MF/SS favoured mogamulizumab over vorinostat across all time points	[68]
Mogamulizumab in Combination with Durvalumab or Tremelimumab in Patients with Advanced Solid Tumors: A Phase I Study.	Dmitriy Zamarin et al.	2021	Multicenter, phase I, dose-escalation study	There is no clear correlation of clinical response with peripheral or intratumoral reduction in CCR4^+^ eTregs or with the baseline degree of CCR4^+^ expression	[100]
Reduced-intensity haploidentical peripheral blood stem cell transplantation using low-dose thymoglobulin for aggressive adult T-cell leukaemia/lymphoma patients in non-complete remission	Makoto Hirosawa et al.	2020	Prospective study	CR was achieved in all the patients after transplantation, including one with pretransplant mogamulizumab therapy; however, T-cell receptor repertoire diversities were low even 1 year after transplantation in next-generation sequencing	[101]
Exposure-Response Analysis for Mogamulizumab in Adults with Cutaneous T-Cell Lymphoma	Mayumi Mukai et al.	2020	Registrational clinical trial	No variable was found to impact efficacy or safety, indicating that there is no need to modify the dose on the basis of this parameter	[102]

## Data Availability

No new data were created or analyzed in this study. Data sharing is not applicable to this article.

## References

[1] Willemze R., Cerroni L., Kempf W., Berti E., Facchetti F., Swerdlow S.H., Jaffe E.S. (2019). The 2018 update of the WHO-EORTC classification for primary cutaneous lymphomas. Blood.

[2] Dai J., Duvic M. (2023). Cutaneous T-Cell Lymphoma: Current and Emerging Therapies. Oncology.

[3] Pileri A., Patrizi A., Agostinelli C., Neri I., Sabattini E., Bacci F., Piccaluga P.P., Pimpinelli N., Pileri S.A. (2011). Primary cutaneous lymphomas: A reprisal. Semin. Diagn. Pathol..

[4] Pileri A., Morsia E., Zengarini C., Torre E., Goteri G., Quaglino P., Pimpinelli N., Paulli M., Pileri S.A., Zinzani P.L. (2023). Epidemiology of cutaneous T-cell lymphomas: State of the art and a focus on the Italian Marche region. Eur. J. Dermatol..

[5] Latzka J. (2023). EORTC consensus recommendations for the treatment of mycosis fungoides/Sézary syndrome—Update 2023. Eur. J. Cancer.

[6] Cerroni L. (2018). Mycosis fungoides-clinical and histopathologic features, differential diagnosis, and treatment. Semin. Cutan Med. Surg..

[7] Trautinger F., Eder J., Assaf C., Bagot M., Cozzio A., Dummer R., Gniadecki R., Klemke C.-D., Ortiz-Romero P.L., Papadavid E. (2017). European Organisation for Research and Treatment of Cancer consensus recommendations for the treatment of mycosis fungoides/Sézary syndrome—Update 2017. Eur. J. Cancer.

[8] Miyashiro D., Sanches J.A. (2023). Mycosis fungoides and Sézary syndrome: Clinical presentation, diagnosis, staging, and therapeutic management. Front. Oncol..

[9] Quadri I., Reneau J.C., Hanel W., Chung C.G. (2023). Advancements in the treatment of mycosis fungoides and Sézary syndrome: Monoclonal antibodies, immunotherapies, and Janus kinase inhibitors. Front. Immunol..

[10] Laghi A., Franceschini C., Mandel V.D., Teoli M., Musicco F., Sansone M., La Malfa A.M., Ardigò M. (2022). Topical chlormethine gel in the treatment of mycosis fungoides: A single-center real-life experience and systematic review of the literature. Dermatol. Ther..

[11] Zinzani P.L., Quaglino P., Pimpinelli N., Berti E., Baliva G., Rupoli S., Martelli M., Alaibac M., Borroni G., Chimenti S. (2006). Prognostic factors in primary cutaneous B-cell lymphoma: The Italian Study Group for Cutaneous Lymphomas. J. Clin. Oncol..

[12] Zinzani P.L., Bonthapally V., Huebner D., Lutes R., Chi A., Pileri S. (2016). Panoptic clinical review of the current and future treatment of relapsed/refractory T-cell lymphomas: Peripheral T-cell lymphomas. Crit. Rev. Oncol. Hematol..

[13] Zinzani P.L., Quaglino P., Violetti S.A., Cantonetti M., Goteri G., Onida F., Paulli M., Rupoli S., Barosi G., Pimpinelli N. (2021). Critical concepts and management recommendations for cutaneous T-cell lymphoma: A consensus-based position paper from the Italian Group of Cutaneous Lymphoma. Hematol. Oncol..

[14] Quaglino P., Maule M., Prince H.M., Porcu P., Horwitz S., Duvic M., Talpur R., Vermeer M., Bagot M., Guitart J. (2019). Global patterns of care in advanced stage mycosis fungoides/Sezary syndrome: A multicenter retrospective follow-up study from the Cutaneous Lymphoma International Consortium. Ann. Oncol..

[15] Kamijo H., Miyagaki T. (2021). Mycosis Fungoides and Sézary Syndrome: Updates and Review of Current Therapy. Curr. Treat. Options Oncol..

[16] Sanches J.A., Cury-Martins J., Abreu R.M., Miyashiro D., Pereira J. (2021). Mycosis fungoides and Sézary syndrome: Focus on the current treatment scenario. An. Bras. Dermatol..

[17] Stuver R., Geller S. (2023). Advances in the treatment of mycoses fungoides and Sézary syndrome: A narrative update in skin-directed therapies and immune-based treatments. Front. Immunol..

[18] Guglielmo A., Zengarini C., Agostinelli C., Sabattini E., Pileri A. (2024). The Role of Cytokines in Cutaneous T-cell Lymphoma: Focus on the State of the Art and Possible Therapeutic Targets. Cells.

[19] Zinzani P.L., Baliva G., Magagnoli M., Bendandi M., Modugno G., Gherlinzoni F., Orcioni G.F., Ascani S., Simoni R., Pileri S.A. (2000). Gemcitabine Treatment in Pretreated Cutaneous T-Cell Lymphoma: Experience in 44 Patients. J. Clin. Oncol..

[20] Marchi E., Alinari L., Tani M., Stefoni V., Pimpinelli N., Berti E., Pagano L., Bernengo M.G., Zaja F., Rupoli S. (2005). Gemcitabine as frontline treatment for cutaneous T-cell lymphoma: Phase II study of 32 patients. Cancer.

[21] Welfringer-Morin A., Bataille P., Drummond D., Bellon N., Ingen-Housz-Oro S., Bonigen J., Schmartz S., Giraud-Kerleroux L., Moulin F., De Saint Blanquat L. (2023). Comparison of idiopathic and drug-induced epidermal necrolysis in children. Br. J. Dermatol..

[22] Akpek G., Koh H.K., Bogen S., O’Hara C., Foss F.M. (1999). Chemotherapy with etoposide, vincristine, doxorubicin, bolus cyclophosphamide, and oral prednisone in patients with refractory cutaneous T-cell lymphoma. Cancer.

[23] Molin L., Thomsen K., Volden G., Aronsson A., Hammar H., Hellbe L., Wantzin G.L., Roupe G. (1987). Oral retinoids in mycosis fungoides and Sézary syndrome: A comparison of isotretinoin and etretinate. A study from the Scandinavian Mycosis Fungoides Group. Acta Derm. Venereol..

[24] Hughes C.F.M., Khot A., McCormack C., Lade S., Westerman D.A., Twigger R., Buelens O., Newland K., Tam C., Dickinson M. (2015). Lack of durable disease control with chemotherapy for mycosis fungoides and Sézary syndrome: A comparative study of systemic therapy. Blood.

[25] Yagi H., Seo N., Ohshima A., Itoh T., Itoh N., Horibe T., Yoshinari Y., Takigawa M., Hashizume H. (2006). Chemokine receptor expression in cutaneous T cell and NK/T-cell lymphomas: Immunohistochemical staining and in vitro chemotactic assay. Am. J. Surg. Pathol..

[26] Patil K., Kuttikrishnan S., Khan A.Q., Ahmad F., Alam M., Buddenkotte J., Ahmad A., Steinhoff M., Uddin S. (2022). Molecular pathogenesis of Cutaneous T cell Lymphoma: Role of chemokines, cytokines, and dysregulated signaling pathways. Semin. Cancer Biol..

[27] Maj J., Jankowska-Konsur A.M., Hałoń A., Woźniak Z., Plomer-Niezgoda E., Reich A. (2015). Expression of CXCR4 and CXCL12 and their correlations to the cell proliferation and angiogenesis in mycosis fungoides. Postep. Dermatol. Alergol..

[28] Miyagaki T., Sugaya M., Murakami T., Asano Y., Tada Y., Kadono T., Okochi H., Tamaki K., Sato S. (2011). CCL11–CCR3 Interactions Promote Survival of Anaplastic Large Cell Lymphoma Cells via ERK1/2 Activation. Cancer Res..

[29] Förster R., Schubel A., Breitfeld D., Kremmer E., Renner-Müller I., Wolf E., Lipp M. (1999). CCR7 coordinates the primary immune response by establishing functional microenvironments in secondary lymphoid organs. Cell.

[30] Narducci M.G., Scala E., Bresin A., Caprini E., Picchio M.C., Remotti D., Ragone G., Nasorri F., Frontani M., Arcelli D. (2006). Skin homing of Sézary cells involves SDF-1-CXCR4 signaling and down-regulation of CD26/dipeptidylpeptidase IV. Blood.

[31] Notohamiprodjo M., Segerer S., Huss R., Hildebrandt B., Soler D., Djafarzadeh R., Buck W., Nelson P.J., von Luettichau I. (2005). CCR10 is expressed in cutaneous T-cell lymphoma. Int. J. Cancer.

[32] Wang Z., Ma J., Zhang H., Ramakrishna R., Mintzlaff D., Mathes D.W., Pomfret E.A., Lucia M.S., Gao D., Haverkos B.M. (2023). CCR4-IL2 bispecific immunotoxin is more effective than brentuximab for targeted therapy of cutaneous T-cell lymphoma in a mouse CTCL model. FEBS Open Bio.

[33] Tuzova M., Richmond J., Wolpowitz D., Curiel-Lewandrowski C., Chaney K., Kupper T., Cruikshank W. (2015). CCR4^+^ T cell recruitment to the skin in mycosis fungoides: Potential contributions by thymic stromal lymphopoietin and interleukin-16. Leuk. Lymphoma.

[34] Kunicki M.A., Amaya Hernandez L.C., Davis K.L., Bacchetta R., Roncarolo M.-G. (2018). Identity and Diversity of Human Peripheral Th and T Regulatory Cells Defined by Single-Cell Mass Cytometry. J. Immunol..

[35] Santagata S., Ieranò C., Trotta A.M., Capiluongo A., Auletta F., Guardascione G., Scala S. (2021). CXCR4 and CXCR7 Signaling Pathways: A Focus on the Cross-Talk Between Cancer Cells and Tumor Microenvironment. Front. Oncol..

[36] Yoshie O., Matsushima K. (2015). CCR4 and its ligands: From bench to bedside. Int. Immunol..

[37] Kim C.H., Rott L., Kunkel E.J., Genovese M.C., Andrew D.P., Wu L., Butcher E.C. (2001). Rules of chemokine receptor association with T cell polarization in vivo. J. Clin. Investig..

[38] Freeman C.M., Stolberg V.R., Chiu B.-C., Lukacs N.W., Kunkel S.L., Chensue S.W. (2006). CCR4 Participation in Th Type 1 (Mycobacterial) and Th Type 2 (Schistosomal) Anamnestic Pulmonary Granulomatous Responses. J. Immunol..

[39] Matsuo K., Nagakubo D., Komori Y., Fujisato S., Takeda N., Kitamatsu M., Nishiwaki K., Quan Y.-S., Kamiyama F., Oiso N. (2018). CCR4 Is Critically Involved in Skin Allergic Inflammation of BALB/c Mice. J. Investig. Dermatol..

[40] Araujo-Pires A.C., Vieira A.E., Francisconi C.F., Biguetti C.C., Glowacki A., Yoshizawa S., Campanelli A.P., Trombone A.P.F., Sfeir C.S., Little S.R. (2015). IL-4/CCL22/CCR4 Axis Controls Regulatory T-Cell Migration That Suppresses Inflammatory Bone Loss in Murine Experimental Periodontitis. J. Bone Miner. Res..

[41] Scheu S., Ali S., Ruland C., Arolt V., Alferink J. (2017). The C-C Chemokines CCL17 and CCL22 and Their Receptor CCR4 in CNS Autoimmunity. Int. J. Mol. Sci..

[42] Mikhak Z., Strassner J.P., Luster A.D. (2013). Lung dendritic cells imprint T cell lung homing and promote lung immunity through the chemokine receptor CCR4. J. Exp. Med..

[43] Kempf W., Mitteldorf C. (2021). Cutaneous T-cell lymphomas-An update 2021. Hematol. Oncol..

[44] Cowan R.A., Scarisbrick J.J., Zinzani P.L., Nicolay J.P., Sokol L., Pinter-Brown L., Quaglino P., Iversen L., Dummer R., Musiek A. (2021). Efficacy and safety of mogamulizumab by patient baseline blood tumour burden: A post hoc analysis of the MAVORIC trial. J. Eur. Acad. Dermatol. Venereol..

[45] Cui L.-Y., Chu S.-F., Chen N.-H. (2020). The role of chemokines and chemokine receptors in multiple sclerosis. Int. Immunopharmacol..

[46] Geller S., Hollmann T.J., Horwitz S.M., Myskowski P.L., Pulitzer M. (2020). C-C chemokine receptor 4 expression in CD8^+^ cutaneous T-cell lymphomas and lymphoproliferative disorders, and its implications for diagnosis and treatment. Histopathology.

[47] Sakamoto Y., Ishida T., Masaki A., Takeshita M., Iwasaki H., Yonekura K., Tashiro Y., Ito A., Kusumoto S., Iida S. (2022). Clinicopathological significance of CD28 overexpression in adult T-cell leukemia/lymphoma. Cancer Sci..

[48] Ishida T., Ueda R. (2006). CCR4 as a novel molecular target for immunotherapy of cancer. Cancer Sci..

[49] Davies M.N., Bayry J., Tchilian E.Z., Vani J., Shaila M.S., Forbes E.K., Draper S.J., Beverley P.C.L., Tough D.F., Flower D.R. (2009). Toward the Discovery of Vaccine Adjuvants: Coupling In Silico Screening and In Vitro Analysis of Antagonist Binding to Human and Mouse CCR4 Receptors. PLoS ONE.

[50] Robles O., Jackson J.J., Marshall L., Talay O., Chian D., Cutler G., Diokno R., Hu D.X., Jacobson S., Karbarz E. (2020). Novel Piperidinyl-Azetidines as Potent and Selective CCR4 Antagonists Elicit Antitumor Response as a Single Agent and in Combination with Checkpoint Inhibitors. J. Med. Chem..

[51] Yokoyama K., Ishikawa N., Igarashi S., Kawano N., Masuda N., Hattori K., Miyazaki T., Ogino S., Orita M., Matsumoto Y. (2008). Potent CCR4 antagonists: Synthesis, evaluation, and docking study of 2,4-diaminoquinazolines. Bioorg. Med. Chem..

[52] Nakagami Y., Kawase Y., Yonekubo K., Nosaka E., Etori M., Takahashi S., Takagi N., Fukuda T., Kuribayashi T., Nara F. (2010). RS-1748, a Novel CC Chemokine Receptor 4 Antagonist, Inhibits Ovalbumin-Induced Airway Inflammation in Guinea Pigs. Biol. Pharm. Bull..

[53] RAPT Therapeutics, Inc. (2023). A Phase 2 Study to Evaluate the Efficacy and Safety of RPT193 as Monotherapy in Adults with Moderate-to-Severe Atopic Dermatitis.

[54] Procopiou P.A., Barrett J.W., Barton N.P., Begg M., Clapham D., Copley R.C.B., Ford A.J., Graves R.H., Hall D.A., Hancock A.P. (2013). Synthesis and structure-activity relationships of indazole arylsulfonamides as allosteric CC-chemokine receptor 4 (CCR4) antagonists. J. Med. Chem..

[55] Kindon N., Andrews G., Baxter A., Cheshire D., Hemsley P., Johnson T., Liu Y.-Z., McGinnity D., McHale M., Mete A. (2017). Discovery of AZD-2098 and AZD-1678, Two Potent and Bioavailable CCR4 Receptor Antagonists. ACS Med. Chem. Lett..

[56] Bozza S., Iannitti R.G., Pariano M., Renga G., Costantini C., Romani L., Bayry J. (2021). Small Molecule CCR4 Antagonists Protect Mice from Aspergillus Infection and Allergy. Biomolecules.

[57] Lee C., Han W., Wang X., Velatooru L.R., Ni X. CCR4 Antagonists in Cutaneous T-Cell Lymphoma (CTCL). https://openworks.mdanderson.org/cgi/viewcontent.cgi?article=1005&context=sumexp22.

[58] Watanabe K., Gomez A.M., Kuramitsu S., Siurala M., Da T., Agarwal S., Song D., Scholler J., Rotolo A., Posey A.D. (2023). Identifying highly active anti-CCR4 CAR T cells for the treatment of T-cell lymphoma. Blood Adv..

[59] Roelens M., de Masson A., Andrillon A., Ram-Wolff C., Biard L., Boisson M., Mourah S., Battistella M., Toubert A., Bagot M. (2022). Mogamulizumab induces long-term immune restoration and reshapes tumour heterogeneity in Sézary syndrome. Br. J. Dermatol..

[60] Horwitz S.M., Scarisbrick J.J., Dummer R., Whittaker S., Duvic M., Kim Y.H., Quaglino P., Zinzani P.L., Bechter O., Eradat H. (2021). Randomized phase 3 ALCANZA study of brentuximab vedotin vs physician’s choice in cutaneous T-cell lymphoma: Final data. Blood Adv..

[61] Kim Y.H., Bagot M., Pinter-Brown L., Rook A.H., Porcu P., Horwitz S.M., Whittaker S., Tokura Y., Vermeer M., Zinzani P.L. (2018). Mogamulizumab versus vorinostat in previously treated cutaneous T-cell lymphoma (MAVORIC): An international, open-label, randomised, controlled phase 3 trial. Lancet Oncol..

[62] Beylot-Barry M., Booken N., Weishaupt C., Scarisbrick J., Wu W., Rosen J.-P., Medley M.C. (2023). Impact of blood involvement on efficacy and time to response with mogamulizumab in mycosis fungoides and Sézary syndrome. J. Eur. Acad. Dermatol. Venereol..

[63] Zhou Y., Vedantham P., Lu K., Agudelo J., Carrion R., Nunneley J.W., Barnard D., Pöhlmann S., McKerrow J.H., Renslo A.R. (2015). Protease inhibitors targeting coronavirus and filovirus entry. Antiviral. Res..

[64] Sato T., Iwase M., Miyama M., Komai M., Ohshima E., Asai A., Yano H., Miki I. (2013). Internalization of CCR4 and Inhibition of Chemotaxis by K777, a Potent and Selective CCR4 Antagonist. Pharmacology.

[65] Ajram L., Begg M., Slack R., Cryan J., Hall D., Hodgson S., Ford A., Barnes A., Swieboda D., Mousnier A. (2014). Internalization of the chemokine receptor CCR4 can be evoked by orthosteric and allosteric receptor antagonists. Eur. J. Pharmacol..

[66] Hawkins N., Muszbek N., Evans R., Dequen-O’Byrne P., Jones T., McNamara L. (2022). Adjusting for treatment crossover in the MAVORIC trial: Survival in advanced mycosis fungoides and Sézary syndrome. J. Comp. Eff. Res..

[67] Hong D.S., Rixe O., Chiu V.K., Forde P.M., Dragovich T., Lou Y., Nayak-Kapoor A., Leidner R., Atkins J.N., Collaku A. (2022). Mogamulizumab in Combination with Nivolumab in a Phase I/II Study of Patients with Locally Advanced or Metastatic Solid Tumors. Clin. Cancer Res..

[68] Porcu P., Hudgens S., Horwitz S., Quaglino P., Cowan R., Geskin L., Beylot-Barry M., Floden L., Bagot M., Tsianakas A. (2021). Quality of Life Effect of the Anti-CCR4 Monoclonal Antibody Mogamulizumab Versus Vorinostat in Patients with Cutaneous T-cell Lymphoma. Clin. Lymphoma Myeloma Leuk..

[69] Zhang Y., Wu Y., Qi H., Xiao J., Gong H., Zhang Y., Xu E., Li S., Ma D., Wang Y. (2017). A new antagonist for CCR4 attenuates allergic lung inflammation in a mouse model of asthma. Sci. Rep..

[70] Herschhorn A., Gu C., Espy N., Richard J., Finzi A., Sodroski J.G. (2014). A broad HIV-1 inhibitor blocks envelope glycoprotein transitions critical for entry. Nat. Chem. Biol..

[71] Marshall L.A., Marubayashi S., Jorapur A., Jacobson S., Zibinsky M., Robles O., Hu D.X., Jackson J.J., Pookot D., Sanchez J. (2020). Tumors establish resistance to immunotherapy by regulating Treg recruitment via CCR4. J. Immunother. Cancer.

[72] Jorapur A., Marshall L.A., Jacobson S., Xu M., Marubayashi S., Zibinsky M., Hu D.X., Robles O., Jackson J.J., Baloche V. (2022). EBV+ tumors exploit tumor cell-intrinsic and -extrinsic mechanisms to produce regulatory T cell-recruiting chemokines CCL17 and CCL22. PLoS Pathog..

[73] Allen S., Newhouse B., Anderson A.S., Fauber B., Allen A., Chantry D., Eberhardt C., Odingo J., Burgess L.E. (2004). Discovery and SAR of trisubstituted thiazolidinones as CCR4 antagonists. Bioorg. Med. Chem. Lett..

[74] Scarisbrick J.J., Prince H.M., Vermeer M.H., Quaglino P., Horwitz S., Porcu P., Stadler R., Wood G.S., Beylot-Barry M., Pham-Ledard A. (2015). Cutaneous Lymphoma International Consortium Study of Outcome in Advanced Stages of Mycosis Fungoides and Sézary Syndrome: Effect of Specific Prognostic Markers on Survival and Development of a Prognostic Model. J. Clin. Oncol..

[75] Sugiyama D., Nishikawa H., Maeda Y., Nishioka M., Tanemura A., Katayama I., Ezoe S., Kanakura Y., Sato E., Fukumori Y. (2013). Anti-CCR4 mAb selectively depletes effector-type FoxP3^+^ CD4^+^ regulatory T cells, evoking antitumor immune responses in humans. Proc. Natl. Acad. Sci. USA.

[76] Beygi S., Duran G.E., Fernandez-Pol S., Rook A.H., Kim Y.H., Khodadoust M.S. (2022). Resistance to mogamulizumab is associated with loss of CCR4 in cutaneous T-cell lymphoma. Blood.

[77] Ohuchi K., Amagai R., Kambayashi Y., Asano Y., Fujimura T. (2022). Serum CCL22 Increased in Advanced Melanoma Patients with Liver Metastases: Report of 5 Cases. Case Rep. Oncol..

[78] Hisamoto T., Suga H., Kawana Y., Oka T., Miyagaki T., Sugaya M., Sato S. (2021). A case of mycosis fungoides successfully treated with combination of bexarotene and mogamulizumab. Dermatol. Ther..

[79] Teoli M., Mandel V.D., Franceschini C., Saraceni P.L., Cicini M.P., Ardigò M. (2022). Mogamulizumab and bexarotene are a promising association for the treatment of advanced cutaneous T-cell lymphomas: A case series. Eur. Rev. Med. Pharmacol. Sci..

[80] Fong S., Hong E.K., Khodadoust M.S., Li S., Hoppe R.T., Kim Y.H., Hiniker S.M. (2021). Low-Dose Total Skin Electron Beam Therapy Combined With Mogamulizumab for Refractory Mycosis Fungoides and Sézary Syndrome. Adv. Radiat. Oncol..

[81] Rubio-Muniz C.A., Sánchez-Velázquez A., Arroyo-Andrés J., Agud-de Dios M., Tarín-Vicente E.J., Falkenhain-López D., Ortiz-Romero P.L. (2024). Mogamulizumab combined with extracorporeal photopheresis for the treatment of refractory mycosis fungoides and Sézary syndrome. Report of seven cases. J. Eur. Acad. Dermatol. Venereol..

[82] Raval N.S., Snowden C.K., De Monnin K.S., Yokoyama C.C., Choi J., Mehta-Shah N., Rosman I.S., Pavlisin J., Musiek A.C. (2021). Scarring alopecia developing after mogamulizumab-associated rash. Eur. J. Dermatol..

[83] Musiek A.C.M., Rieger K.E., Bagot M., Choi J.N., Fisher D.C., Guitart J., Haun P.L., Horwitz S.M., Huen A.O.-L., Kwong B.Y. (2022). Dermatologic Events Associated with the Anti-CCR4 Antibody Mogamulizumab: Characterization and Management. Dermatol. Ther..

[84] Mitteldorf C., Langer N., Kempf W., Schön M.P. (2023). Mogamulizumab-associated rash simulating lupus miliaris disseminatus faciei. J. Eur. Acad. Dermatol. Venereol..

[85] Martínez-Fernández S., Suh-Oh H.-J., Couselo-Rodríguez C., Soto-García D., Álvarez C., Flórez Á. (2023). Mogamulizumab-associated rash: A challenging case report and literature review. Australas. J. Dermatol..

[86] Kincaid C.M., Sharma A.N., Lee B.A., Pinter-Brown L.C., Smith J., Linden K., Mesinkovska N.A. (2023). Alopecia areata-like presentations with mogamulizumab therapy. JAAD Case Rep..

[87] Breen I.D., Brumfiel C.M., Patel M.H., Rosenthal A.C., Rule W.G., DiCaudo D.J., Craig F.E., Pittelkow M.R., Mangold A.R. (2021). Mogamulizumab-induced interface dermatitis drug rash treated successfully with methotrexate and extracorporeal photopheresis in a patient with Sézary syndrome. JAAD Case Rep..

[88] Caruso L., Castellino A., Dessì D., Flenghi L., Giordano A., Ibatici A., Massone C., Pileri A., Proietti I., Pupo L. (2022). Italian Real-Life Experience on the Use of Mogamulizumab in Patients with Cutaneous T-Cell Lymphomas. Cancer Manag. Res..

[89] Wang J.Y., Hirotsu K.E., Neal T.M., Raghavan S.S., Kwong B.Y., Khodadoust M.S., Brown R.A., Novoa R.A., Kim Y.H., Rieger K.E. (2020). Histopathologic Characterization of Mogamulizumab-associated Rash. Am. J. Surg. Pathol..

[90] Hirotsu K.E., Neal T.M., Khodadoust M.S., Wang J.Y., Rieger K.E., Strelo J., Hong E., Kim Y.H., Kwong B.Y. (2021). Clinical Characterization of Mogamulizumab-Associated Rash During Treatment of Mycosis Fungoides or Sézary Syndrome. JAMA Dermatol..

[91] Pileri A., Clarizio G., Zengarini C., Casadei B., Sabattini E., Agostinelli C., Zinzani P.L. (2023). Mogamulizumab-associated rashes, their presentation and prognostic significance: A single-centre retrospective case series analysis. J. Eur. Acad. Dermatol. Venereol..

[92] Barta S.K., Liu N., DerSarkissian M., Chang R., Ye M., Duh M.S., Surinach A., Fanale M., Yu K.S. (2023). Real-World Treatment Patterns and Clinical Outcomes With Brentuximab Vedotin or Other Standard Therapies in Patients With Previously Treated Cutaneous T-Cell Lymphoma in the United States. Clin. Lymphoma Myeloma Leuk..

[93] Fujikawa K., Saito T., Kurose K., Kojima T., Funakoshi T., Sato E., Kakimi K., Iida S., Doki Y., Oka M. (2023). Integrated analysis of phase 1a and 1b randomized controlled trials; Treg-targeted cancer immunotherapy with the humanized anti-CCR4 antibody, KW-0761, for advanced solid tumors. PLoS ONE.

[94] Izutsu K., Makita S., Nosaka K., Yoshimitsu M., Utsunomiya A., Kusumoto S., Morishima S., Tsukasaki K., Kawamata T., Ono T. (2023). An open-label, single-arm phase 2 trial of valemetostat for relapsed or refractory adult T-cell leukemia/lymphoma. Blood.

[95] Saito T., Kurose K., Kojima T., Funakoshi T., Sato E., Nishikawa H., Nakajima J., Seto Y., Kakimi K., Iida S. (2021). Phase Ib study on the humanized anti-CCR4 antibody, KW-0761, in advanced solid tumors. Nagoya J. Med. Sci..

[96] Maeda Y., Wada H., Sugiyama D., Saito T., Irie T., Itahashi K., Minoura K., Suzuki S., Kojima T., Kakimi K. (2021). Depletion of central memory CD8^+^ T cells might impede the antitumor therapeutic effect of Mogamulizumab. Nat. Commun..

[97] Horwitz S., Zinzani P.L., Bagot M., Kim Y.H., Moskowitz A.J., Porcu P., Dwyer K., Sun W., Herr F.M., Scarisbrick J. (2021). Lack of impact of type and extent of prior therapy on outcomes of mogamulizumab therapy in patients with cutaneous T cell lymphoma in the MAVORIC trial. Leuk. Lymphoma.

[98] Dubois S.P., Miljkovic M.D., Fleisher T.A., Pittaluga S., Hsu-Albert J., Bryant B.R., Petrus M.N., Perera L.P., Müller J.R., Shih J.H. (2021). Short-course IL-15 given as a continuous infusion led to a massive expansion of effective NK cells: Implications for combination therapy with antitumor antibodies. J. Immunother. Cancer.

[99] Rolf D., Elsayad K., Eich H.T. (2021). Acute and sub-acute toxicity profile of ultra-hypofractionated low-dose total skin electron beam with two 4 Gy fractions for cutaneous T cell lymphoma. J. Cancer Res. Clin. Oncol..

[100] Zamarin D., Hamid O., Nayak-Kapoor A., Sahebjam S., Sznol M., Collaku A., Fox F.E., Marshall M.A., Hong D.S. (2020). Mogamulizumab in Combination with Durvalumab or Tremelimumab in Patients with Advanced Solid Tumors: A Phase I Study. Clin. Cancer Res..

[101] Hirosawa M., Yamaguchi T., Tanaka A., Kominato Y., Higashi T., Morimoto H., Tsukada J. (2020). Reduced-intensity haploidentical peripheral blood stem cell transplantation using low-dose thymoglobulin for aggressive adult T cell leukemia/lymphoma patients in non-complete remission. Ann. Hematol..

[102] Mukai M., Mould D., Maeda H., Narushima K., Greene D. (2020). Exposure-Response Analysis for Mogamulizumab in Adults With Cutaneous T-Cell Lymphoma. J. Clin. Pharmacol..

[103] Matsuo K., Hatanaka S., Kimura Y., Hara Y., Nishiwaki K., Quan Y.S., Kamiyama F., Oiso N., Kawada A., Kabashima K. (2019). A CCR4 antagonist ameliorates atopic dermatitis-like skin lesions induced by dibutyl phthalate and a hydrogel patch containing ovalbumin. Biomed. Pharmacother..

[104] Bissonnette R., DuBois J., Facheris P., Del Duca E., Kim M., Correa Da Rosa J., Trujillo D.L., Bose S., Pagan A.D., Wustrow D. (2023). Clinical and molecular effects of oral CCR4 antagonist RPT193 in atopic dermatitis: A Phase 1 study. Allergy.

[105] Ndao M., Nath-Chowdhury M., Sajid M., Marcus V., Mashiyama S.T., Sakanari J., Chow E., Mackey Z., Land K.M., Jacobson M.P. (2013). A cysteine protease inhibitor rescues mice from a lethal Cryptosporidium parvum infection. Antimicrob. Agents Chemother..

[106] Beck T.C., Beck K.R., Holloway C.B., Hemings R.A., Dix T.A., Norris R.A. (2020). The C-C Chemokine Receptor Type 4 Is an Immunomodulatory Target of Hydroxychloroquine. Front. Pharmacol..

[107] Shukla L., Ajram L.A., Begg M., Evans B., Graves R.H., Hodgson S.T., Lynn S.M., Miah A.H., Percy J.M., Procopiou P.A. (2016). 2,8-Diazaspiro[4.5]decan-8-yl)pyrimidin-4-amine potent CCR4 antagonists capable of inducing receptor endocytosis. Eur. J. Med. Chem..

[108] Raymondi Silva J., Iftinca M., Fernandes Gomes F.I., Segal J.P., Smith O.M.A., Bannerman C.A., Silva Mendes A., Defaye M., Robinson M.E.C., Gilron I. (2022). Skin-resident dendritic cells mediate postoperative pain via CCR4 on sensory neurons. Proc. Natl. Acad. Sci. USA.

[109] Solari R., Pease J.E. (2015). Targeting chemokine receptors in disease—A case study of CCR4. Eur. J. Pharmacol..

[110] Bogacka J., Pawlik K., Ciapała K., Ciechanowska A., Mika J. (2022). CC Chemokine Receptor 4 (CCR4) as a Possible New Target for Therapy. Int. J. Mol. Sci..

[111] Purandare A.V., Wan H., Somerville J.E., Burke C., Vaccaro W., Yang X., McIntyre K.W., Poss M.A. (2007). Core exploration in optimization of chemokine receptor CCR4 antagonists. Bioorg. Med. Chem. Lett..

[112] Yamamoto S., Matsuo K., Nagakubo D., Higashiyama S., Nishiwaki K., Oiso N., Kawada A., Yoshie O., Nakayama T. (2018). A CCR4 antagonist enhances DC activation and homing to the regional lymph node and shows potent vaccine adjuvant activity through the inhibition of regulatory T-cell recruitment. J. Pharmacol. Sci..

[113] Cahn A., Hodgson S., Wilson R., Robertson J., Watson J., Beerahee M., Hughes S.C., Young G., Graves R., Hall D. (2013). Safety, tolerability, pharmacokinetics and pharmacodynamics of GSK2239633, a CC-chemokine receptor 4 antagonist, in healthy male subjects: Results from an open-label and from a randomised study. BMC Pharmacol. Toxicol..

[114] Smith D.A., Beaumont K., Maurer T.S., Di L. (2018). Relevance of Half-Life in Drug Design. J. Med. Chem..

[115] Jin C., Gao B.-B., Zhou W.-J., Zhao B.-J., Fang X., Yang C.-L., Wang X.-H., Xia Q., Liu T.-T. (2022). Hydroxychloroquine attenuates autoimmune hepatitis by suppressing the interaction of GRK2 with PI3K in T lymphocytes. Front. Pharmacol..

[116] Gadhe C.G., Kim M. (2015). Insights into the binding modes of CC chemokine receptor 4 (CCR4) inhibitors: A combined approach involving homology modelling, docking, and molecular dynamics simulation studies. Mol. BioSyst..

[117] Fauzi Y.R., Nakahata S., Chilmi S., Ichikawa T., Nueangphuet P., Yamaguchi R., Nakamura T., Shimoda K., Morishita K. (2021). Antitumor effects of chloroquine/hydroxychloroquine mediated by inhibition of the NF-κB signaling pathway through abrogation of autophagic p47 degradation in adult T-cell leukemia/lymphoma cells. PLoS ONE.

[118] Wang C., Reusser N., Shelton M., Reed J., Doan H., Torres-Cabala C.A., Dabaja B., Duvic M. (2015). An unusual case of cytotoxic peripheral T-cell lymphoma. JAAD Case Rep..

[119] Heyl J., Mehregan D., Kado J., Campbell M. (2014). A case of idiopathic follicular mucinosis treated with bexarotene gel. Int. J. Dermatol..

[120] Schneider S.W., Metze D., Bonsmann G. (2010). Treatment of so-called idiopathic follicular mucinosis with hydroxychloroquine. Br. J. Dermatol..

[121] Nair R., Westin J. (2020). CAR T-Cells. Adv. Exp. Med. Biol..

[122] Perera L.P., Zhang M., Nakagawa M., Petrus M.N., Maeda M., Kadin M.E., Waldmann T.A., Perera P.-Y. (2017). Chimeric antigen receptor modified T cells that target chemokine receptor CCR4 as a therapeutic modality for T-cell malignancies. Am. J. Hematol..

